# Comparative Analysis of Non-Coding RNA Transcriptomics in Heart Failure

**DOI:** 10.3390/biomedicines10123076

**Published:** 2022-11-30

**Authors:** Carlos Garcia-Padilla, Estefanía Lozano-Velasco, Virginio Garcia-Lopez, Amelia Aranega, Diego Franco, Virginio Garcia-Martinez, Carmen Lopez-Sanchez

**Affiliations:** 1 Department of Human Anatomy and Embryology, Faculty of Medicine, Institute of Molecular Pathology Biomarkers, University of Extremadura, 06006 Badajoz, Spain; 2 Department of Experimental Biology, University of Jaen, 23071 Jaen, Spain; 3 Medina Foundation, 18016 Granada, Spain

**Keywords:** heart failure, transcriptomics, non-coding RNA, microRNA, long non-coding RNA, circular RNA

## Abstract

Heart failure constitutes a clinical complex syndrome with different symptomatic characteristics depending on age, sex, race and ethnicity, among others, which has become a major public health issue with an increasing prevalence. One of the most interesting tools seeking to improve prevention, diagnosis, treatment and prognosis of this pathology has focused on finding new molecular biomarkers since heart failure relies on deficient cardiac homeostasis, which is regulated by a strict gene expression. Therefore, currently, analyses of non-coding RNA transcriptomics have been oriented towards human samples. The present review develops a comparative study emphasizing the relevance of microRNAs, long non-coding RNAs and circular RNAs as potential biomarkers in heart failure. Significantly, further studies in this field of research are fundamental to supporting their widespread clinical use. In this sense, the various methodologies used by the authors should be standardized, including larger cohorts, homogeneity of the samples and uniformity of the bioinformatic pipelines used to reach stratification and statistical significance of the results. These basic adjustments could provide promising steps to designing novel strategies for clinical management of patients with heart failure.

## 1. Introduction

Despite significant efforts made to improve prevention, diagnosis and treatment of cardiovascular diseases, these pathologies remain responsible for high morbidity and mortality worldwide [1,2]. Particularly, heart failure (HF) is a major public health issue with an increasing prevalence and high rate of hospitalization, readmission and outpatient visits, thus producing a considerable burden on the health care system [3,4,5,6].

HF represents a specific cardiomyopathy with various symptomatic features associated with individual patient conditions. Apart from that, there is a large number of risk factors including ischemic heart disease, myocardial fibrosis, hypertension, smoking, obesity and diabetes which can help to predict incidence and severity of HF [3,4].

Based on the left ventricular ejection fraction (LVEF), the most used parameter for diagnosis, treatment and prognosis of HF [7,8,9,10,11,12,13], three categories have been described: (i) heart failure with reduced ejection fraction (HFrEF) defined as LVEF < 40%; (ii) HF with mid-range EF (HFmrEF) defined as LVEF ranging between 40 and 49% (EF borderline), and (iii) HF with preserved EF (HFpEF), LVEF ≥50%. HFrEF is more prevalent in young male patients, with high incidence of coronary artery diseases and hypertension. HFpEF—which displays a prevalence of 50% of the global HF patients—is more common in female patients and more advanced age. The causes of those differences remain unclear. HFmrEF—with a prevalence of 10–25%—determines an intermediate clinical entity between HFrEF and HFpEF [14,15,16]. Furthermore, HF diagnosis has been widely supported by means of sensitive markers [17] such as B-type natriuretic peptide (BNP) and N-terminal pro-brain natriuretic peptide (NT-proBNP). Since HF presents a high incidence among the population, it is essential to find new molecular biomarkers which may constitute crucial criteria for prevention, diagnosis, treatment and prognosis in this type of cardiac diseases.

From a molecular point of view, HF relies, at least in part, on the loss of cardiac homeostasis, which is governed by tight gene expression regulation [18,19]. This mechanism is controlled by RNA molecules: messenger RNAs (mRNAs), encoding proteins, and non-coding RNAs (ncRNAs), lacking protein-coding potential.

Non-coding RNAs have been traditionally considered as a non-functional part of the genome. However, currently ncRNAs have demonstrated to play an essential role in biological and physiological processes, as well as in many diseases [20,21,22,23,24,25]. According to the number of nucleotides and characteristics [26,27,28,29,30], ncRNAs are classified into (i) small non-coding RNAs (≤200 nucleotides), including microRNAs, snoRNAs, piRNAs, and tRNAs; (ii) long non-coding RNAs (>200 nucleotides), including intronic, enhancer, circular and intergenic lncRNAs, and (iii) circular RNA (circRNA), which lacks free ends and comprises a wide range of ncRNAs. This third emerging class is produced by a non-canonical splicing event (back-splicing), a process in which a downstream splice-donor site is covalently linked to an upstream splice-acceptor site.

Currently, numerous research papers have been focused on the aforementioned molecular factors and their relationship with heart function and specific cardiac pathologies. It is well known that the capacity of heart function depends mostly of proper cardiomyocytes structure and function [31]. Defects on cardiomyocytes biology are translated in loss-of-function capacity of heart pumping [32,33]. Hemodynamic stress and low oxygen levels trigger a cardiomyocyte ventricular hypertrophy as adaptive response, which has a compensatory role to enhance cardiac performance and diminish ventricular wall tension and oxygen consumption [34]. In this hypertrophy context, cardiomyocytes increase in cell size, enhance protein synthesis, and heighten organization of the sarcomere [35,36]. Although the hypertrophic response is beneficial in the short term, its maintenance over time leads to the progression of cardiac hypertrophy towards symptomatic heart failure. The molecular bases that govern hypertrophy response are very complex, including both coding RNAs and non-coding RNAs as pivotal modulators [37]. The coding RNA roles have been widely described, while the impact of non-coding RNAs has begun to be addressed in the last decade [38,39,40,41]. MicroRNAs modulate gene expression at post-transcriptional level by recognizing and binding to targets mRNA and triggers their degradation. As result of modulation mediated by microRNAs, molecular pathways involved in cardiac hypertrophy could be activated or repressed. In hypertrophy context, several microRNAs have been analysed as regulators of hypertrophy-related pathways such as inflammation, redox or Ca^2+^ signaling [38]. The mechanism of lncRNA roles is more complex than that of microRNAs due to the fact that lncRNAs could exert their function both at transcriptional and post-transcriptional levels, interacting with all types of RNA molecules, proteins and different chromatic modulators. Several lncRNAs have been described in a pro-hypertrophy context, such as H19, CTBP1-AS1, ROR and CHAST, as well as in an antihypertrophic context—MHRT, TINCR, Lnc-Plscr4, XIST and CYTOR—in distinct animal models and human [39,42,43,44,45,46,47,48,49,50]. Finally, circRNAs act binding to protein or microRNAs interfering with them and blocking their function. To date, all hypertrophy-related circRNAs have been reported acting as sponge of several microRNAs involved in hypertrophy cascade signaling [41,51,52]. In particular, CircSlc8a1 can bind to miR-133, thus avoiding SRF recognizing, inducing cardiac hypertrophy. Finally, although just a few cases of peptides generated by circRNAs have been reported, Circ-ZNF609 has demonstrated to modulate myoblast proliferation in a possible cardiac regeneration [51,52].

In this review, we will establish a comparative analysis of ncRNA transcriptomics in order to highlight their influence in HF.

## 2. Transcriptomic Analysis in Heart Failure

**Figure 1:**
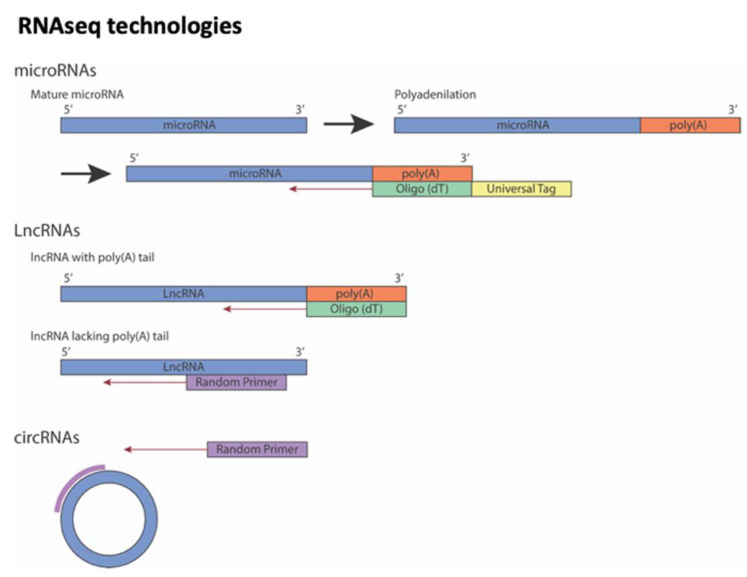
Overview of strategies to sequencing different non-coding RNAs. Note that lncRNAs could be sequencing using Oligo (dT) or Random Primer depending on whether poly(A)-tail on lncRNA is present or not.

Transcriptomic analysis is a comprehensive analysis that provides information about all the RNA transcripts of an organism, including mRNAs and ncRNAs, offering the possibility of measuring gene expression in different developmental stages and physiological/pathological conditions. Transcript studies have been performed for decades. The first approaches used microarrays, in which a large number of genes can be quantitatively detected by using the principle of molecular hybridization [53]. Improved techniques were supported by sequence-based approaches [54,55,56] such as Sanger sequencing technique used for random sequences of individual transcripts from cDNA libraries and tag-based methods, i.e., serial analysis of gene expression (SAGE) or cap analysis gene expression (CAGE). However, these technologies are based on the reliance upon existing knowledge about genome sequence. Additional techniques have been introduced to detect unknown genes or alternatively spliced genes. In this sense, RNA-seq technology, which is based on next-generation sequencing (NGS) and whereby shorter reads, allows to sequence thousands or even millions of cDNA molecules at the same time, helping to understand cell function and metabolic mechanisms [57]. This methodology has been implemented at the single-cell level (single-cell RNA-seq) identifying the transcriptome and other multi-omic features of different cell types. However, single-cell transcriptomic analyses have some technical limitations such as to recognize lncRNAs only with polyA-tail or low expression levels of this kind of RNA molecules (see Figure 1). Furthermore, most studies using the traditional bulk RNA-seq methods cannot fully characterize the intrinsic heterogeneity between individual cells and the complexity of circRNAs at the single-cell level [58,59].

### 2.1. Transcriptional Analysis of microRNAs in Heart Failure

MicroRNAs are on average 20–22 ribonucleotides in length and display the capacity to bind to the 3’ untranslated region (3´UTR) of coding RNAs by complementary base pairing, promoting their degradation and/or translational blockage. The role of microRNAs as post-transcriptional modulators has been widely described in multiple biological processes, including cell development, differentiation, growth and homeostasis [60,61,62,63]. Interestingly, it has been widely described that microRNAs are associated with cardiac physiological and pathological phenotypes, and nowadays circulating microRNAs are being deeply studied as potential HF diagnostic biomarkers.

Particularly, microRNA profiling studies have been carried out in human HF samples, being identified as differentially expressed microRNAs during pathogenesis and progression. In this line, it has been proposed that circulating microRNAs constitute relevant potential biomarkers for the diagnostic of clinical HF [64]. By means of microRNA microarray analyses (GSE53437) obtained from HF patients, in plasma and whole blood, 32 microRNAs (validation performed using RT-qPCR) showed expression levels differing from controls. Among these, 12 dysregulated microRNAs were found to be related to specific HF. Even through some of these are specifically related to HFpEF and some others to HPrEF, this is an interesting biomarker tool to distinguish between both types of HF.

In a later study [65] concerning microRNA microarray analyses (GSE104150) performed from plasma samples, 94 microRNAs were found to be differentially expressed in patients with clinical HF stage C or D (following the World Health Organization/International Society and the Federation of Cardiology criteria), 7 of them significantly upregulated after RT-qPCR validation.

**Figure 2:**
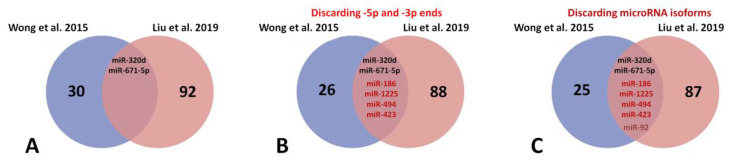
Venn diagram for analysis of commonly deregulated microRNAs related to heart failure in Wong et al., 2015 [64] and Liu et al., 2019 [65] studies: (**A**) mir-320d and miR-671 are commonly deregulated microRNAs in HF; (**B**) if -5p and -3p ends are discarded of the analysis, four new common microRNAs appear to be commonly deregulated; miR-186, miR-1225, miR-494 and miR-423; (**C**) finally, discarding microRNA isoforms of only one new common microRNA, miR-92, is deregulated in HF.

Our comparative analysis (Figure 2) of the above two studies—32 microRNAs (GSE53437) and 94 miRNAs (GSE104150) differentially expressed—presented only two microRNAs in common: miR-320d and miR-671-5p (Figure 2A). When we discarded -5p and -3p ends, four additional microRNAs were observed in common: miR-186, miR-1225, miR-494, and miR-423 (Figure 2B). Finally, when we discarded microRNA isoforms, an extra microRNA appeared in common: miR-92 (Figure 2C). This analysis supports the concept that a limited number of microRNAs are observed in common molecular mechanisms related to HF, while those remaining differentially expressed microRNAs might be attributed to other cofactors such as heterogeneity of the sampling and risk factor index associated with patients.

#### 2.1.1. miR-320d

It has been demonstrated that miR-320d, a member of miR-320 family, plays a potential role in modulating cell growth and apoptosis [66]. In particular, low levels of miR-320d are detected in cardiomyocytes of atrial fibrillation patients, leading to elevated apoptosis and suppressed cell viability [67]. These processes are mediated through the regulation of STAT3, a direct target of miR-320d. Other studies have revealed that the activation of STAT3 mediates structural and electrical remodeling during atrial fibrillation and also contributes to its inflammatory process [68,69].

Although miR-320d is a common microRNA in both studies based on microarray analyses [64,65], additional miR-320 family members have been related to heart diseases that could lead to HF. For example, miR-320a presents high levels in coronary artery disease and modulates serum response factor (SRF) expression, leading to atherogenesis [70]. Notably, miR-320 [71] could also enlarge the cardiac infarct size of ischemia/reperfusion mice by inhibiting heat shock protein 20 (hsp20). In further studies, based on a cellular context, the dual role of miR-320 has been established, that is, its overexpression both promotes cardiac dysfunction in cardiomyocytes and palliates cardiac fibrosis and hypertrophy in cardiac fibroblasts during HF progression [72].

#### 2.1.2. miR-671-5p

A series of different roles have been proposed for miR-671-5p. In HF, miR-671-5p is upregulated, showing its highest expression in HFrEF patients, thus considered as a predictive differential maker in the three HF categories [64]. According to the drug-mRNA-miRNA network, NF-κb and its corresponding miRNA-671-5p have been identified as drug targets of HF [73]. With respect to nuclear factor erythroid 2-related factor 2 (Nrf2), which plays a critical role in regulating cardiac redox status and is able to modulate the expression of several microRNAs in the heart, this factor promotes miR-671-5p expression, inducing several ARE-bearing microRNAs as well as leading to cardiac-specific transcript silencing. These microRNAs are considered “ReductomiR”, working as myocardial reductive stress mediators [74,75]. Furthermore, analyzing the microRNA function in the proatherogenic effects of oxidized phospholipids, miR-671-5p expression, modulated by Nrf2, has been described in human endothelial cells [76].

#### 2.1.3. miR-186

With respect to miR-186, this microRNA has been found upregulated in plasma samples from acute myocardial infarction patients [77,78]. In particular, miR-186-5p has been observed to be involved in acute coronary syndrome by modulating HIF-1 signaling pathway, affecting glucose metabolism as well as hypoxia response [79,80].

#### 2.1.4. miR-1225

A group of microRNAs including miR-1225-5p has been demonstrated to be downregulated in plasma samples obtained from elderly patients with angina [81], which could increase ADAMTS (disintegrin and metalloproteinase with thrombospondin type 1 motifs), a predicted miR-1225-5p target gene that promotes vulnerable plaque formation [82,83]. In this sense, miR-1225-5p is considered as a potential biomarker in cardio- and cerebrovascular diseases [81,84].

#### 2.1.5. miR-494

This microRNA has been associated with a cardioprotective role against ischemia/reperfusion-induced cardiac injury by means of Akt pathway activation [85]. Several authors have reported that miR-494 modulates Wnt signaling pathway through leucine-rich-alpha-2-glycoprotein 1 (LRG1) regulation, inducing proliferation, migration and invasion of vascular endothelial cells and fibroblasts during myocardial infarction [86]. Other studies have described that a reduced miR-494-3p expression is related to myocardial injury in patients with septic shock. In addition, through experimental analyses, these studies have revealed that miR-494-3p overexpression downregulates the phosphatase and tensin homolog (PTEN), thus reducing both synthesis and release of TNF-α and IL-6 in cardiomyocytes, proving their protective physiological functions [87].

#### 2.1.6. miR-423

Within miR-423, it has been reported that miR-423-5p is upregulated in failing human myocardium [88,89]. This particular microRNA can be used as a biomarker to differentiate stable coronary artery disease and acute myocardial infarction. Notably, miR-423-5p is downregulated within the first 24 h and increases after 6 months [90]. Other authors have reported that miR-423-5p plays a significant role in cardiomyocyte apoptosis [91] by modulating m-O-GlcNAc transferase (OGT), caspase 3/7, Bax and c-caspase 3 [92]. In addition, both myocardial infarction and HF generate mitochondrial dysfunction by hypoxia and reoxygenation cycles. These effects can be alleviated by miR-423-5p inhibition, which activates Wnt/β-catenin pathways targeting MYBL2. Finally, other studies have revealed that high levels of circulating miR-423-3p are correlated with lower risk of coronary artery disease [93].

#### 2.1.7. miR-92

Dysregulation of miR-92 family has been related to diverse cardiovascular diseases. In particular, it has been reported that miR-92a is upregulated in diabetes mellitus patients, constituting a potential marker associated with cardiovascular diseases [94]. Other authors have proposed that serum exo-miR-92b-5p, whose expression is increased in patients with dilated cardiomyopathy, could be considered as potential biomarker for acute HF [95].

### 2.2. Transcriptomic Analysis of LncRNAs in Heart Failure

LncRNAs [96,97] are able to function both as transcriptional regulators (modulating nuclear gene expression in different ways, including epigenetic landscape control, transcriptional complex scaffolding and/or decoy molecules) and post-transcriptional regulators (modulating microRNA degradation, mRNA stability and/or protein translation). Notably, several authors have highlighted the pivotal role of lncRNAs in cardiac remodelling and arrhythmogenic pathologies such as dilated cardiomyopathy and atrial fibrillation, respectively, representing HF risk factors [4,19,46,98,99,100]. In this sense, we analyzed a number of research studies based on lncRNA transcriptomic approaches performed in samples from both right and left ventricles belonging to HF patients, as described below.

Transcriptomic analyses carried out by monitoring lncRNA alterations of right ventricle (RV) biopsies from HF patients [101] reported 78 lncRNAs deregulated in comparison with control samples. Interestingly, 48 of those are represented by classical lncRNAs—35 downregulated and 13 upregulated, while the 30 remaining lncRNAs are catalogued as antisense lncRNAs—18 downregulated and 12 upregulated. Among the first set, lncRNA AP00078783.2 is characterized by displaying the lowest expression levels in HF conditions. Moreover, bioinformatic analysis suggests a potential role of this lncRNA as a microRNA decoy molecule for miR-942, miR-580 and miR-4760-3p [102]. In particular, miR-942 has been described as an apoptotic-induced protector in cardiomyocytes. In this sense, by means of *in silico* analysis, interaction between AP00078783.2 and miR-942 has been observed, suggesting a potential role of this lncRNA in apoptosis regulation. With respect to miR-580 and miR-4760-3p, their possible roles in cardiac pathologies have not been sufficiently explored to date.

The second set includes antisense lncRNAs whose expressions are correlated with the neighboring mRNAs expressions sharing promotor and chromatic features. Within the 30 deregulated antisense lncRNAs [101], NPPA-AS1 is characterized by displaying high expression in the HF right ventricle compared to the residual levels found in control samples. Notably, NPPA gene expression is considered as a relevant biomarker in early stages of different cardiac diseases, including HF [103]. Similarly to NPPA-AS1, NPPA expression is enhanced in ventricles as a response to cardiac injuries. For this reason, deeper research studies oriented to molecular interaction between both genes are necessary.

Several authors [37] have studied left ventricle (LV) appendices to explore possible changes in a transcriptomic environment since this ventricle is the chamber that suffers the most intense remodeling as a consequence of HF. Two transcriptomic analyses of LV samples in HF patients have uncovered multiple lncRNA deregulations as response to this pathological process. Others studies [104] have reported differential expression of lncRNAs from LV biopsies of HF induced by ischemic dilated cardiomyopathy. It was observed that 13 lncRNAs were deregulated with respect to control samples—3 upregulated and 10 downregulated. Furthermore, 9 of them—CDKN2B-AS1/ANRIL, EGOT, H19, HOTAIR, LOC285194/TUSC7, RMRP, RNY5, SOX2-OT and SRA1—were detected in blood samples from HF patients, highlighting their potential roles as biomarkers of this disease. By means of Gene Ontology (GO) analysis, these 9 lncRNAs show an association with two biological processes, insulin signaling pathway and cell cycle, which are altered in HF.

Another transcriptomic GO analysis of LV samples from HF patients [105] uncovered two additional lncRNAs: AC018647.1 and AC009113.1. Differential expressions of both lncRNAs were detected in three independent transcriptomic datasets. Moreover, competing endogenous RNA (ceRNA) network analysis showed an interrelation between AC018647.1 and AC009113.1, with 170 and 149 associated genes, respectively. ceRNA network analysis suggests that OR51E1 gene is strongly correlated with AC018647.1, whereas RAB9B gene is linked to AC009113.1. Additionally, these two genes have been proposed as modulators of mitochondrial metabolism in HF [106,107].

Finally, in order to assess possible interactions between lncRNAs and mRNAs in an HF context, several authors [108] performed a comprehensive transcriptomic analysis of LV samples from HF. Out of the 993 deregulated lncRNAs found, only 66 of them showed a correlation with a subset of mRNAs. Among those 66 lncRNAs, Neat1 was the most significant, located in the central node of the ceRNA network, displaying the best score. Neat1 expression has also been noticed in cardiac fibrosis and HF, appointed as a relevant biomarker in cardiac disease diagnosis [108,109].

**Figure 3:**
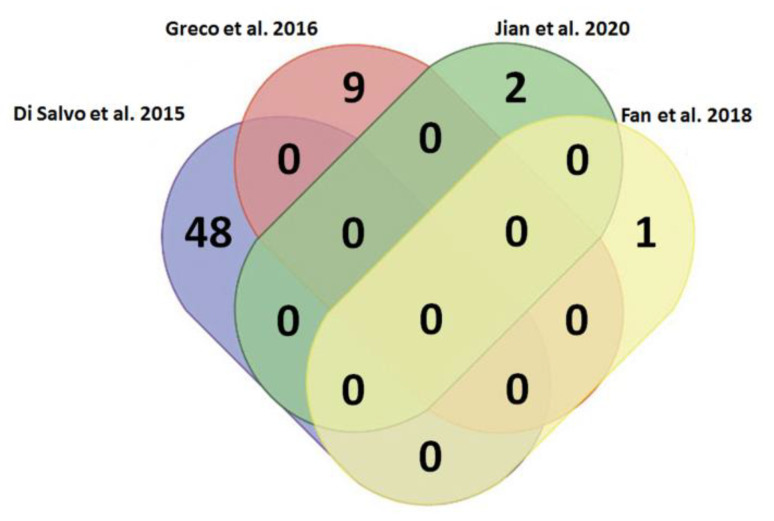
Venn diagram for the analysis of commonly deregulated lncRNAs related to heart failure in Di Salvo et al., 2015 [101], Greco et al., 2016 [104], Jian et al., 2020 [105] and Fan et al., 2018 [108]. It has been evidenced that several lncRNAs are deregulated in HF; however, there are not any commonly deregulated among the four studies considered in this review.

In this review, we sought to identify if there were any shared lncRNAs as potential biomarkers in HF. The four above studies under analysis did not present any lncRNAs in common (Figure 3). Several reasons could justify these findings: (i) the different regional areas selected by the authors; (ii) the chosen samples obtained from RV vs. LV; (iii) heterogeneous features—age, clinical histories and cardiovascular risk factors—within the cohorts; (iv) variability on the bioinformatic pipelines used to reach stratification and statistical significance of the results, and (v) insufficient sampling data from the transcriptomic analyses available in HF.

### 2.3. Transcriptomic Analysis of Circular RNAs in Heart Failure

**Figure 4:**
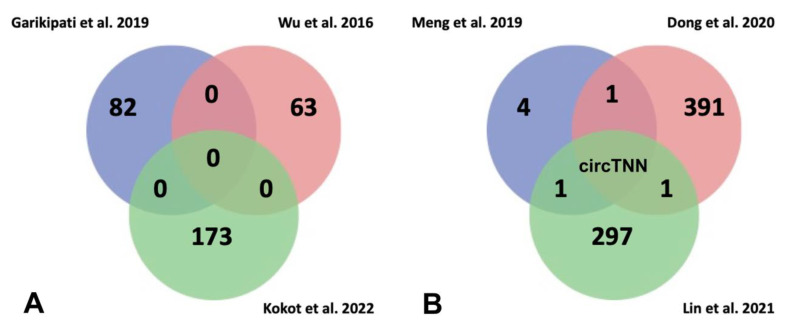
(**A**) Venn diagram for analysis of commonly deregulated circRNAs related to heart failure in Garikipati et al., 2019 [117], Wu et al., 2016 [118], and Kokot et al., 2022 [119] studies. It has been evidenced that several circRNAs are deregulated in HF; however, there are not any commonly deregulated circRNAs among the three studies considered in this review. (**B**) Venn diagram for analysis of commonly deregulated circRNAs related to risk factors to heart failure in Meng et al., 2019 [120], Dong et al., 2020 [121] and Lin et al., 2021 [122]. The comparison between them showed that circTTN is deregulated in all studies suggesting a role of possible biomarker of HF.

Circular RNAs (circRNA) display differential expression profiles among species, development stages, and pathologies. Their lack of free ends grants them higher stability in comparison with linear transcripts, becoming attractive candidates as biomarkers and therapeutic tools. Although circRNAs have been described as non-coding RNAs, new discoveries might challenge such consideration, since increasing numbers of studies have found that circRNAs contain open reading frames that can be translated in a cap-independent manner such as internal ribosome entry site (IRES) and N6-methyladenosine (m6A). Furthermore, some peptides generated by circRNAs translation exert physiological function in several tumors such as digestive system neoplasms [28,29,110]. Currently, evidence has been reported on the role of circRNAs in several human diseases, including diabetes mellitus, neurological disorders, chronic inflammatory processes, as well as cardiovascular pathologies [41,111,112,113,114,115,116]. The differential expression profiles of circRNAs have been studied in myocardial infarction-induced HF in mice [117,118]; the main results have been summarized in Figure 4.

Several authors have analysed circRNA transcriptomics from myocardial infarction (MI) hearts after 3 days following left anterior descending coronary artery ligation in mice, detecting 82 circRNAs deregulated (41 upregulated and 41 downregulated) compared to sham hearts [117]. By means of functional assays, those authors highlighted circFnd3 which attenuates post-MI LV dysfunction modulating positively the endothelial cell function, reducing cardiomyocyte apoptosis and cardiac fibrosis. Furthermore, circFnd3 increased angiogenesis and thus promoted oxygen supply to heart. Although in previous studies [118] 63 differentially expressed circRNAs (29 upregulated and 34 downregulated) were identified, no common circRNAs were detected between both studies. Most recently [119] it has been demonstrated that adenosine-to-inosine (A-to-I) RNA editing is responsible for 80% of the RNA editing events in human myocardium. Notably, failing hearts display reduced RNA editing, mediated by ADAR2 downregulation which binds to RNA regions and modulates stability of double-stranded RNA and Alu elements. Loss of stability of Alu elements on double-stranded RNA enhance recirculation of pre-mRNAs, resulting in newly formed circRNAs. Deep transcriptomic analysis from failing left ventricle have highlighted misregulation of 173 circRNAs (166 upregulated and 7 downregulated) where the most of upregulated circRNAs are associated with reduced RNA editing in the host gene (Figure 4A). Finally, these authors analyzed the functional role of one of them in hiPSC-CM, circAKAP13, demonstrating that it is essential for sarcomere regularity.

Furthermore, circRNA transcriptomic analyses [120,121,122,123] have also been reported in HF risk factor models: (i) in cardiomyocyte hypertrophy, induced by high glucose levels, identifying five differentially expressed circRNAs, and (ii) in dilated cardiomyopathy in two assays performed on human patients, first identifying 392 differentially expressed circRNAs (101 upregulated and 291 downregulated) and second 298 differentially expressed circRNAs (213 upregulated and 85 downregulated). When we compared the results of the above works, confluence among them was unappreciated. Only circTTN was identified in two different studies performed in samples from failure human hearts and dilated human hearts, suggesting that this circRNA could be a possible risk biomarker for heart failure (Figure 4B). Therefore, further research should be carried out to consolidate circRNAs as potential HF biomarkers.

## 3. Conclusions and Perspectives

Heart failure has become a significant cardiovascular pathology with a vast social and economic impact. Today, a full understanding of HF etiopathogenesis constitutes a biomedical challenge. In particular, a large number of recent studies have oriented their research towards transcriptional regulators, addressing cardiovascular diseases. Currently, there is scientific evidence that distinct post-transcriptional mechanisms, particularly those orchestrated by non-coding RNAs, govern key molecular pathways with clear impact in multiple cardiovascular disorders. Therefore, mapping and unraveling the functional role of these ncRNAs is essential to providing novel biomarkers and further understanding HF clinical behavior.

### 3.1. ncRNAs Transcriptomic Analyses as Biomarkers

In this work, we have searched for common differentially expressed ncRNAs between those transcriptomic analyses from HF patients published to date, aiming to gain insight into deregulated ncRNAs and their impact both on prognosis and HF disease course. In this sense, this study sought to identify possible ncRNAs as biomarkers for clinical applications in order to minimize those pathology effects that characterize this disease. The scarce evidence of common ncRNA found among the different transcriptomic analyses under study suggests that molecular bases underlying HF pathology present high complexity, requiring further research.

As efficient molecular techniques, transcriptomic analyses provide an entry point to dissecting impaired gene expressions. Although several studies have uncovered the differential expression of ncRNAs in HF, as reported in this review, to date, ncRNA (microRNAs, lncRNAs and circRNAs) have still limited validity for clinical use as biomarkers in HF. Several reasons could justify the above statement. First, most studies have relatively small cohorts to verify the diagnostic and prognostic potential of ncRNAs in HF. Second, insufficient non-coding panel screens have been listed, making it difficult to select the most differentially expressed ncRNAs. Third, the unstandardized methodology used in those studies under analysis hinders understanding of the respective results obtained. Fourth, since some microRNAs are expressed in a time- and stage-specific manner, the expression pattern of dysregulated ncRNAs is not always constant and may fluctuate with time. The wide range of sampling revised sources together with the physiological variability and genetic background of the patients under study could justify the discrepancy between the different transcriptomic analyses.

### 3.2. ncRNAs Transcriptomic Analyses in Therapy

Clearly, the impact of ncRNAs as cardiac function modulators as well as their deregulation in several cardiovascular diseases and associated processes (such as cardiomyocyte apoptosis and cardiac fibrosis) appoint these ncRNAs as potential therapeutic molecules. However, our transcriptomic analyses show that misregulation of ncRNA transcriptomic is dependent not only on a specific cardiac disease, but also the physiological variability and genetic background of each patient, hindering the development of a therapeutic molecular signature to treat cardiac dysfunction.

Consequently, further studies in this research field are required in order to better understand transcriptional regulators, which could provide promising steps to designing novel strategies to heal a damaged heart.

## Data Availability

Not applicable.
